# Nutrition Screening Tools and the Prediction of Clinical Outcomes among Chinese Hospitalized Gastrointestinal Disease Patients

**DOI:** 10.1371/journal.pone.0159436

**Published:** 2016-08-04

**Authors:** Fang Wang, Wei Chen, Kay Stearns Bruening, Sudha Raj, David A. Larsen

**Affiliations:** 1 Nutrition Department of Peking Union Medical College Hospital, Beijing, China; 2 Parenteral and enteral Department of Peking Union Medical College Hospital, Beijing, China; 3 Syracuse University Department of Public Health, Food Studies and Nutrition, Syracuse, New York, United States of America; University Hospital Llandough, UNITED KINGDOM

## Abstract

Nutrition risk Screening 2002 (NRS-2002) and Subjective Global Assessment (SGA) are widely used screening tools but have not been compared in a Chinese population. We conducted secondary data analysis of a cross-sectional study which included 332 hospitalized gastrointestinal disease patients, collected by the Gastrointestinal department of Peking Union Medical College Hospital (PUMCH) in 2008. Results of NRS-2002 and SGA screening tools, complications, length of stay (LOS), cost, and death were measured. The agreement between the tools was assessed via Kappa (κ) statistics. The performance of NRS-2002 and SGA in predicting LOS and cost was assessed via linear regression. The complications and death prediction of tools was assessed using receiver operating characteristic (ROC) curves. NRS-2002 and SGA identified nutrition risk at 59.0% and 45.2% respectively. Moderate agreement (κ >0.50) between the two tools was found among all age groups except individuals aged ≤ 20, which only slight agreement was found (κ = 0.087). NRS-2002 (R square 0.130) and SGA (R square 0.140) did not perform differently in LOS prediction. The cost prediction of NRS-2002 (R square 0.198) and SGA (R square 0.190) were not significantly different. There was no difference between NRS-2002 (infectious complications: area under ROC (AUROC) = 0.615, death: AUROC = 0.810) and SGA (infectious complications: AUROC = 0.600, death: AUROC = 0.846) in predicting infectious complication and death, but NRS-2002 (0.738) seemed to perform better than SGA (0.552) in predicting non-infectious complications. The risk of malnutrition among patients was high. NRS-2002 and SGA have similar capacity to predict LOS, cost, infectious complications and death, but NRS-2002 performed better in predicting non-infectious complications.

## Background

Malnutrition occurs when energy imbalance either results from overnutrition or undernutrition. Undernutrition results from nutrients and energy deficiencies due to decreased intake or increased demand. Undernutrition is a common problem in hospitals and other health care centers, with prevalence ranging from 10% to 60% among patients [1–4]. In gastrointestinal wards prevalence of malnutrition tends toward the higher range due to the difficulties of food intake, impaired function of digestion and absorption caused by gastrointestinal diseases. In addition to burdening clinical units, malnutrition is related to poor clinical outcomes for patients, including longer length of stay (LOS), increased cost, increased risk of complications, and a higher death rate [5–8]. Effective nutrition intervention decreases the risk of poor clinical outcomes due to malnutrition [5,6]. Conversely, unnecessary nutrition support results in wasted medical resources and increased burden on health care providers and patients [9]. As the pre-step of the nutrition care process (NCP), nutrition screening aims to detect the presence of and the risk of developing undernutrition, allowing dietitians to provide nutrition care for patients at risk in order to improve outcomes. A good screening tool with high clinical outcome predictive validity is positively correlated with clinical outcomes. However, there is no gold standard for nutrition risk screening.

NRS-2002 and SGA are two screening tools in wide spread use. They assess a patient’s risk of malnutrition depending upon weight and dietary changes, current disease state, and physical examination (waist circumference, edema, muscle wasting, etc.). NRS-2002, introduced by the European Society for Parenteral and Enteral Nutrition (ESPEN), has been applied and reported among Chinese patients [10]. SGA application is not as well studied and comparisons between SGA and NRS-2002 among Chinese patients are limited.

The purpose of this study is to assess prevalence of nutrition risk determined by NRS-2002 and SGA among patients in a Chinese gastrointestinal ward, and then compare the agreement between these two screening tools. Additionally, this study examines the clinical outcome predicting capacity of NRS-2002 and SGA for the outcomes of cost, LOS, complication, and death. Findings could benefit health care providers to choose the most appropriate screening tool for gastrointestinal inpatients.

## Methods

### Participants

The clinical investigation was carried out in the gastrointestinal ward. The original data were collected by clinical dietitians in Peking Union Medical College Hospital (PUMCH), a referral and teaching hospital including more than 2000 beds.

Patients were included in the study if they met the following criteria: 1) gastroenterology patients aged between 18 and 90 years old; 2) scheduled to stay at least one night in hospital; 3) no surgery planned before 8 a.m. on the following day; 4) voluntary participation; 5) capable to communicate for interviewing. Patients with less than one day LOS and surgery planned for the following day were excluded due to the limited time for screening. Patients who were sent to the intensive care unit directly or had a critical illness were not included in the study because the patients could not be interviewed for nutrition screening. Women who were pregnant, provided breast feeding, or had given birth within the past six months were also excluded.

In 2008, 334 participants admitted to the GI department met the inclusion criteria and were included in the study (350 patients were invited to participate). Two subjects were excluded from data analysis due to missing data. For convenience, age groups were broken down into cohorts of 15 years starting from the age of 20, i.e. ≤20, 21–35, 36–50, 51–65, 66–80, and ≥81 year old groups.

### Screening

Nutrition risk screening was performed within 24 hours of admission using both NRS-2002 and SGA conducted by the same dietitian. Simultaneously, anthropometric parameters (height, fasting weight, BMI), age, and the diagnosis were recorded for each patient. After screening, participants with malnutrition or at risk of malnutrition determined by either NRS-2002 or SGA were referred to clinical dietitians for further nutritional care using the NCP.

NRS-2002 classifies patients as at risk of malnutrition on a scale of 1–6. SGA classifies patients as nourished (A), mildly and moderately malnourished (B), or severely malnourished (C) relying on the evaluation about weight and dietary changes, gastrointestinal symptoms and functional problems. Patients were classified as at nutrition risk with a score of 3 or above by the NRS-2002 and with a score of B (mildly and moderately malnourished) or C (malnourished) by SGA.

### Clinical outcomes

LOS, incidence of complications, cost, and death were selected as indicators of clinical outcomes because they are influenced by nutrition status, and reflect the effectiveness and efficiency of medical and nutritional intervention [9]. Infectious complications among these patients included new onset of wound infection, abdominal infection, perianal abscess, tuberculosis, systemic inflammatory response syndrome (SIRS), positive culture test, pneumonia, urinary tract infection, abdominal fistula infection, pressure sores, fungal infection, and oral infections. Non-infectious complications among these patients included new onset of anemia, myocardial infarction, upper gastrointestinal bleeding, and organ failure. The clinical outcome information was collected from the patients’ medical record. The dietitians continued to visit patients and collected the outcome indicators until patient discharge.

### Analysis

The prevalence of nutrition risk detected by NRS-2002 and SGA was calculated for the total patient population and for categorized age groups. Cohen’s kappa (κ) was run to assess the chance-corrected agreement between NRS-2002 and SGA for classifying nutrition risk with 95% confidence intervals (CI). The results were interpreted as follows: <0, no agreement; 0 to 0.19, poor agreement; 0.20 to 0.39, fair agreement; 0.40 to 0.59, moderate agreement; 0.60 to 0.79, substantial agreement; and 0.80 to 1.00, almost perfect agreement [11].

Linear regression was used to assess the performance of NRS-2002 and SGA in predicting LOS and cost. NRS-2002 and SGA were included in the regressions as a linear variable. Five patients who died during hospitalization were excluded from LOS analysis, so LOS was assessed for 327 subjects. LOS and cost were not normally distributed, so the outcomes were log-transformed. Due to a limited number of subjects aged ≤20 and ≥81, age was categorized as four groups: ≤35, 36–50, 51–65, ≥ 66. Age was regarded as a categorical variable because the relationship between age and the outcomes was assumed to be nonlinear. Gender and presence of infectious and non-infectious complications were included as covariates. To determine differences in the predicting capacity of NRS-2002 or SGA for different age groups, a global f-test was used to compare a full model for LOS and cost with a saturated model including the interaction between NRS-2002/ SGA and categorized age. This test compares the log likelihood of the full model (no interaction between screening tool and age) to the log likelihood of the saturated model (interaction between screening tool and age. The accuracy of NRS-2002 and SGA was compared through R-square for each model.

Receiver operating characteristic (ROC) curves [12,13] were applied to assess the predicting capacity of NRS-2002 and SGA for infectious, non-infectious complications, and death using the previously described regression model. Specificity and sensitivity were measured from the ROC curve analysis, and accuracy of the diagnostic test was evaluated by the area under ROC curve. The results were interpreted as follows: 0.9–1, excellent; 0.80–0.90, good; 0.70–0.80, fair; 0.60–0.70, poor; 0.50–0.60, inadequate.

For all analyses, statistical significance was set at *P*≤0.05. Adjustment was not made for the number of hypothesis tests, which could potentially inflate the probability of type 1 error. Data was analyzed by the statistical package IBM SPSS Statistics (IBM Corp., USA) version 20 for Windows. The Peking University Medical College Hospital Institutional Review Board approved the study protocol in 2008 (Registration No. S-203), and all participants were provided written informed consent. The present secondary data analysis was deemed exempt by the Institutional Review Board of Syracuse University in 2013 (IRB# 13–283). Data are available in S1 Dataset.

## Results

A total of 332 patients (99.4% of total patients recruited) were analyzed in the study. Of these patients, 141 (42.5%) were female and 191 (57.5%) were male. Ages ranged from 18 to 86 years old. The mean age was 53 years (SD 18 years), and median age was 47 years. Among patients, 14 subjects were aged ≤ 20, 54 subjects were aged 21–35, 68 subjects were aged 36–50, 94 subjects were aged 51–65, 90 subjects were aged 66–80, and 12 subjects were aged ≥ 80 years.

Five patients died (1.5%) during the hospitalization and were removed from the LOS analysis, resulting in 327 patients. The mean LOS from admission to discharge was 23.6 days (SD 19.4 days). The mean cost of hospitalization was 25,106.00 CNY (4,030.51 USD) (SD 30,985.46 CNY, 4,976.23 USD). Detailed clinical outcome data for different age groups are shown in Table 1. Eighty-one (24.4%) patients had infectious complications; sixty (74.1%) and 47 (58.0%) of those with infectious complications were detected as having nutrition risk by NRS-2002 and SGA, respectively. Twelve (3.3%) patients experienced non-infectious complications; nine (75.0%) and five (41.7%) of those with non-infectious complications were classified with nutrition risk by NRS-2002 and SGA, respectively.

**Table 1:** Detailed clinical outcomes for different age groups.

Age group	n	Nutrition risk detected by NRS-2002, n(%)	Nutrition risk detected by SGA, n(%)	LOS (days)	Cost (USD)	Death rate (%)	Infectious Complications (%)	Non-infectious Complications
≤20	14	10 (0.715)	8 (0.571)	31.2	4475.0	1 (7.1)	5 (35.7)	1 (7.1)
21–35	54	40 (0.741)	26 (0.481)	24.8	3801.3	0	35 (64.8)	2 (3.7)
36–50	68	38 (0.559)	28 (0.412)	26.9	4180.2	0	17 (25.0)	0
51–65	94	44 (0.468)	37(0.394)	24.4	4101.9	2 (2.1)	21 (22.3)	2 (2.1)
66–80	90	53 (0.589)	42 (0.467)	18.6	4106.7	2 (2.2)	21 (23.3)	7 (7.8)
81–95	12	11 (0.917)	9 (0.750)	17.6	2564.6	0	3 (25.0)	0
Total	332	196 (0.590)	150 (0.452)	23.6	4030.5	5 (1.5)	81 (24.4)	12 (3.6)

LOS, length of hospital stay; NRS-2002, Nutrition risk Screening 2002; SGA, Subjective Global Assessment.

Cut points: NRS-2002, at nutrition risk when the score is ≥3; SGA, at nutrition risk when the level is B or C.

### The prevalence of nutrition risk

**Fig 1:**
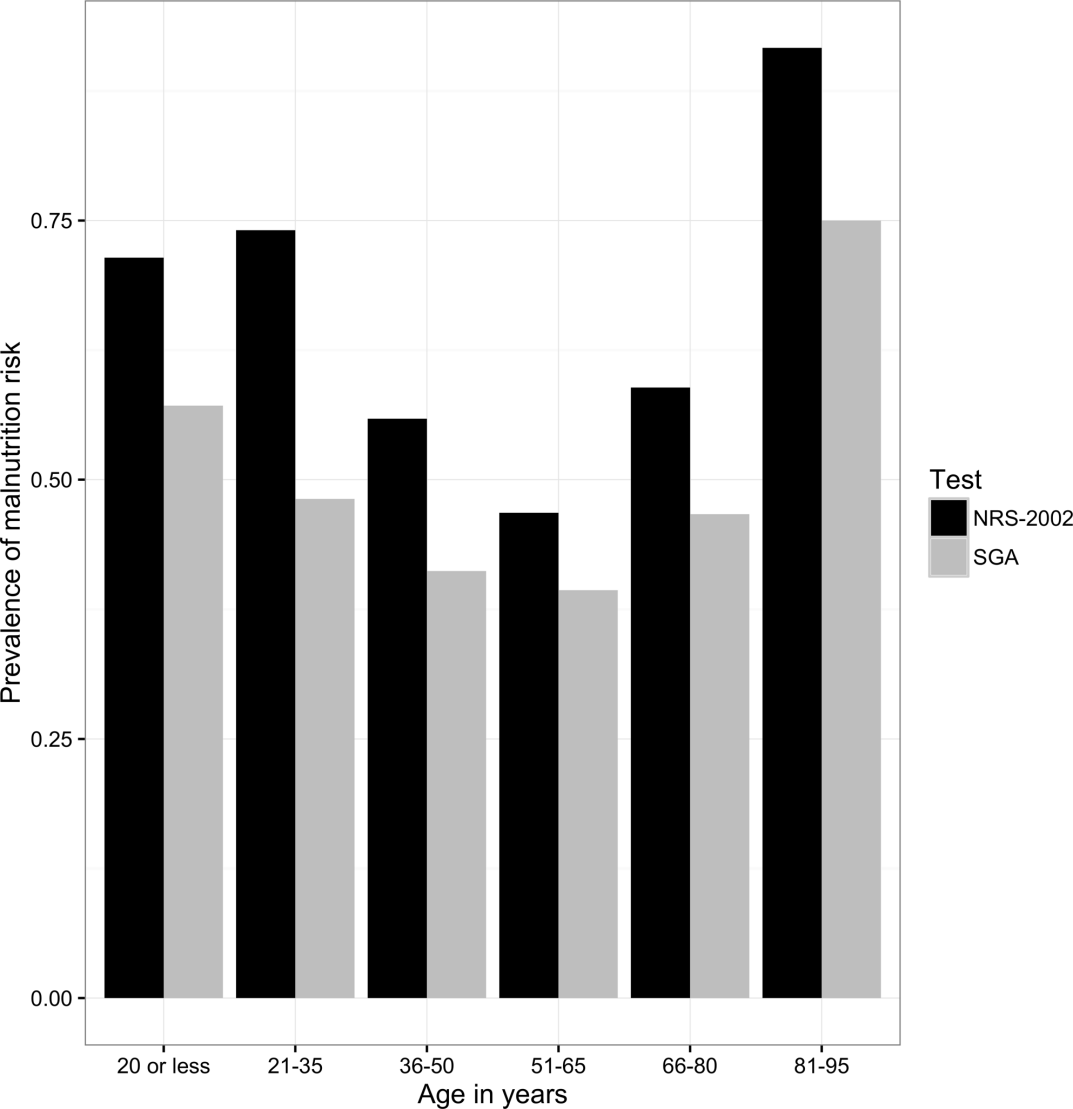
Prevalence of nutritional risk for different age groups. NRS-2002, Nutrition Risk Screening 2002; SGA, Subjective Global Assessment.

The prevalence of nutrition risk among all subjects was 59.0% according to NRS-2002, and 45.2% relying on SGA. Among the different age groups upon admission, the highest prevalence of nutrition risk was in the ≥ 81 years group detected either by NRS-2002 or SGA, and the lowest prevalence of nutrition risk was in 51–65 years group (Fig 1).

### Agreement between NRS-2002 and SGA

There was moderate agreement between the two screening tools, κ = 0.514 (95% CI, 0.428 to 0.604), p<0.0005. When stratifying by age, moderate agreement was observed for all age groups with the exception of individuals aged ≤20 (Table 2).

**Table 2:** Agreement between NRS-2002 and SGA: κ-index.

Age group	n	Nutrition risk detected by NRS-2002 (%)	Nutrition risk detected by SGA (%)	κ-index
≤20	14	71.4	57.1	0.087
21–35	54	74.1	48.1	0.418
36–50	68	55.9	41.2	0.539
51–65	94	46.8	39.4	0.590
66–80	90	58.9	46.7	0.495
81–95	12	91.7	75.0	0.429
total	332	59.0	45.2	0.514

NRS-2002, Nutrition Risk Screening 2002; SGA, Subjective Global Assessment

### Association between nutrition risk screening and clinical outcomes

A one-step increase in malnutrition risk as measured through NRS-2002 or SGA was associated with an increase of 12.7% or 29.6%, respectively in hospitalization cost after adjusting for age and sex of the patient (Table 3). There was little difference between the hospitalization cost predicting capacity of NRS-2002 (R square = 0.198) and SGA (R square = 0.190). A one-step increase in nutrition risk as measured through NRS-2002 or SGA was associated with an increase of 8.6% or 27.2%, respectively in LOS after adjusting for age and gender of the patient (Table 3). There was little difference between the LOS predicting capacity of NRS-2002 (R square = 0.130) and SGA (R square = 0.140). Inclusion of the interaction between the screening tool (NRS-2002 or SGA) and categorized age did not improve the model for the outcomes of LOS and cost (Table 4).

**Table 3:** Relationship between nutrition risk and LOS and cost.

Outcome	assessment	B	t	*p*-value	R square
LOS	NRS-2002	0.086	3.061	0.002	0.130
	SGA	0.272	3.613	0.001	0.140
Cost	NRS-2002	0.127	3.788	0.000	0.198
	SGA	0.296	3.309	0.001	0.190

NRS-2002: Nutrition risk screening 2002; SGA: Subjective global assessment; LOS: Length of stay

**Table 4:** Model comparison and f-test.

Withdrawal assessment	outcome	Sum of square	Degree of freedom	F-test
NRS-2002, Full model	LOS	28.678	7	0.366
NRS-2002, Saturated model	LOS	32.212	10	
SGA, full model	LOS	30.798	7	0.289
SGA, Saturated model	LOS	33.720	10	
NRS-2002, Full model	COST	69.954	7	0.234
NRS-2002, Saturated model	COST	75.229	10	
SGA, full model	COST	67.084	7	0.289
SGA, Saturated model	COST	74.167	10	

Full model included categorized age, gender, infectious and non-infectious complications.

NRS-2002: Nutrition risk screening 2002; SGA: Subjective global assessment; LOS: Length of stay

**Fig 2:**
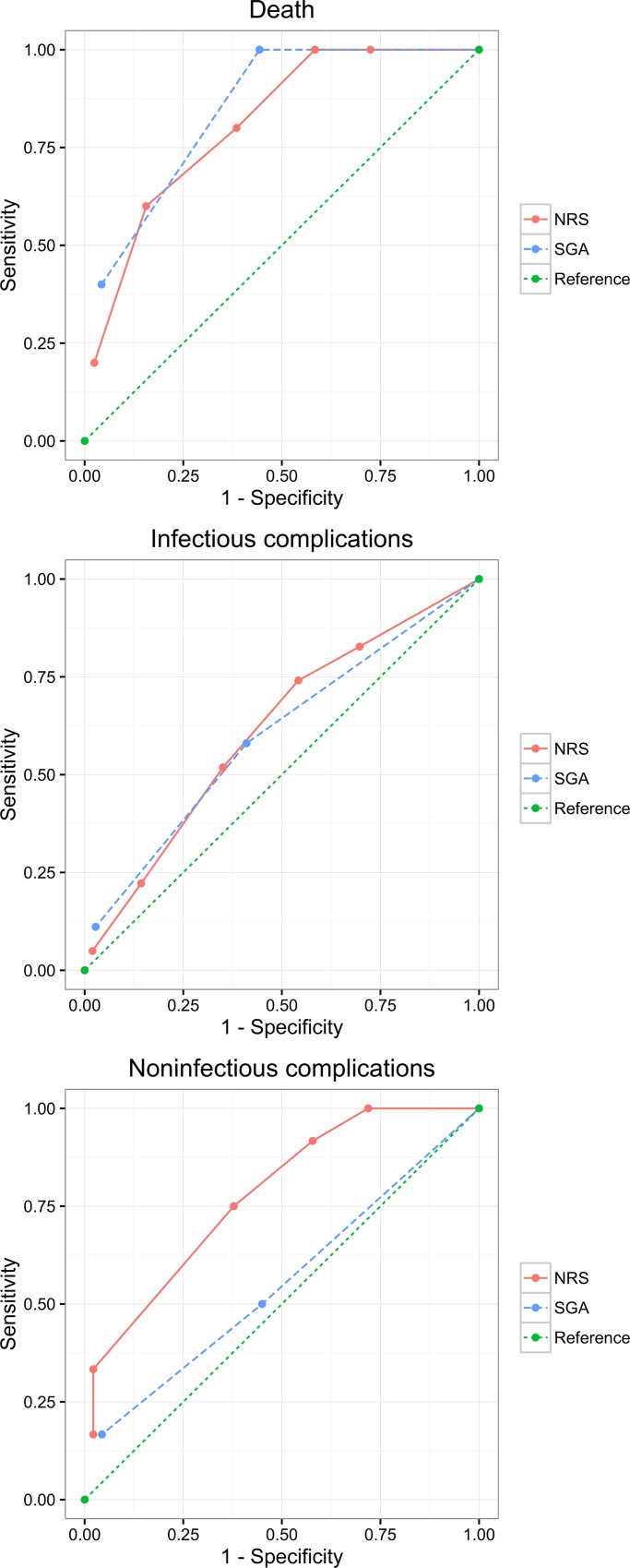
Nutritional screening tools and evaluated clinical outcomes including infectious and non-infectious complications, and death. The most effective tool in predicting unfavorable clinical outcomes is that with the largest area under the receiver operating characteristic curve. NRS-2002, Nutrition risk Screening 2002; SGA, Subjective Global Assessment.

The results of the area under ROC curve analysis showed that the complication and death predicting capacity of NRS-2002 and SGA was generally better than random guessing (Fig 2, Table 5). NRS-2002 and SGA had poor predictive capacity for infectious complications (0.60–0.70), and good predicting capacity for death (0.80–0.90), but they did not perform differently. For non-infectious complications, NRS-2002 had fair predicting capacity (0.70–0.80), whereas SGA had poor predicting capacity (0.50–0.60).

**Table 5:** Clinical outcomes and area under ROC curve values of the two nutritional screening tools according to evaluated outcomes.

Screening tool	Clinical outcomes (area under ROC curve)		
	Infectious complications	Non-infectious complications	Death
NRS-2002	0.615	0.738	0.810
SGA	0.600	0.552	0.846

NRS-2002, Nutrition risk Screening 2002; SGA, Subjective Global Assessment.

## Discussion

### Prevalence of nutrition risk

We conducted a secondary data analysis of nutrition risk as measured through NRS-2002 and SGA as well as the association between the measurements and clinical outcomes, such as LOS, cost, complications and death in a sample of gastrointestinal inpatients at PUMCH. Nutrition risk among patients was high, measured at 59.0% by NRS-2002 and 45.2% by SGA, which was consistent with other studies, but in the upper level of the nutrition risk ranges of 10–60% [1–4,9,14–19]. A possible reason for the high prevalence might be the fact that as a referral hospital, PUMCH dealt with the most difficult diseases, so patients presented with more severe health conditions and higher risks of malnutrition. Comparatively, Johns Hopkins Hospital in the US reported the prevalence of nutrition risk to be 51.0%, with the highest risk being observed in the gastrointestinal department [20]. Gastrointestinal diseases often lead to decreased food intake and impaired function of digestion and absorption. Additionally, the inclusion of patients scheduled for surgery and directed to fast might be another reason for the high prevalence of malnutrition, shown by a Danish study a 57% malnutrition rate in gastro-surgery departments [19].

A higher prevalence of nutrition risk was observed in elder patients (>66 years), which was in agreement with previous studies [21–23]. Age is known to be a powerful contributor to malnutrition development [23], however, a high malnutrition rate was also observed surprisingly among patients ≤ 35 years by both NRS-2002 and SGA, particularly in ≤ 20 years old group. The current study was to assess the prevalence of risk rather than the severity of malnutrition [10]. It is possible the extent of malnutrition among younger adults was lower than elder adults, despite having a high frequency of nutrition risk.

The nutrition risk prevalence detected by NRS-2002 was higher than that detected by SGA for all age groups. SGA focuses more on chronic or established nutrition risk rather than acute nutritional changes compared to NRS-2002, which may explain the discrepancy. For example, the changes of subcutaneous fat and muscles upon physical examination of SGA are long term results of malnutrition, hence acute cases of nutrition risk may not be recognized by SGA [24].

In contradiction to some misgivings about the difficulties in implementing routine use of SGA [25], nearly all patients in this study completed NRS-2002 (99.7%) and SGA (99.4%). Other studies have also found the vast majority of patients capable and willing to complete NRS-2002 and SGA [10,20,26]. NRS-2002 can be completed in a few minutes, but training is required to improve competence using SGA due to its subjective nature. Further studies will assess the amount of time spent on each tool and feedback from patients and dietitians to determine clinician and patient preference.

### Agreement of NRS-2002 and SGA

Moderate agreement was observed between NRS-2002 and SGA for the total population (κ statistic, 0.514), which was similar to results of previous studies conducted by Kyle *et al*. [27] and Velasco *et al*. [23] with κ statistics of 0.480 and 0.620, respectively. The tools showed moderate agreement for individuals at and above 36 years old. However, poor agreement was found for individuals less than 35 years of age, driven by the ≤ 20 year old group with slight agreement. The small number of individuals in the ≤ 20 years category limits the interpretation of the κ statistic and further investigation is needed to determine appropriate malnutrition screening tools in this age group.

### Clinical outcome predicting capacity

The objective of nutrition screening is to accurately identify patients who have higher risk of malnutrition and who could benefit from nutrition therapy. In this study, positive relationships were observed among nutrition risk and clinical outcomes. Patients with higher nutrition risk were more likely to experience more infectious and non-infectious complications, longer LOS, higher cost, and higher mortality. There was no significant interaction between age and NRS-2002 and SGA, suggesting that NRS-2002 or SGA did not perform much differently according to ages. However, the R-square of each model was small, so it was possible the relationship between NRS-2002 or SGA and clinical outcomes was non-linear. The relationships between nutritional status, LOS, and cost are not necessarily causal; rather LOS and cost might be a reflection of the severity of the underlying diseases, economic and even educational status of the patients and their families. In this analysis disease severity was not explicitly included, which may have explained the relatively poor explanatory power of the regression models. The NRS-2002 indirectly includes a measure of disease severity which is one of the parameters used to determine nutrition risk. The explicit inclusion of disease severity greatly complicates modeling approaches with many independent variables all at great risk of colinearity and requiring much larger sample sizes. Some researchers have taken the severity of disease into account, by classifying disease into different levels [9], or just classifying as severe disease and non-severe disease [25], however these measurements were not available in this dataset and could not be included.

The linear regression measuring the association among screening tools and LOS and cost showed that there were not large differences in the predictive capacity of the models with NRS-2002 or SGA for either LOS or cost. Even though compared to NRS-2002, a one-step increase in SGA was associated with a relatively larger increase in cost, the difference was due to having fewer categories in the SGA classification compared to the NRS-2002 classification.

The area under ROC revealed that both NRS-2002 and SGA had generally good predictive capacity for complications (>0.500), especially for death (>0.800). The two screening tools did not perform differently for predicting infectious complications and death, but NRS-2002 was more accurate than SGA to predict non-infectious complications.

It is worth noting that NRS-2002 had identified a higher portion of patients with nutrition risk compared to SGA (59.0%:45.2%), and NRS-2002 was also more efficient than SGA in predicting non-infectious complication. Previous studies conducted by Raslan et al. [16] and Kyle *et al*. [27] indicated that among NRS-2002, SGA, Malnutrition Universal Screening Tool (MUST), and Nutrition risk Index (NRI), NRS-2002 had a good prediction of unfavorable clinical outcomes despite finding the lowest rate of nutrition risk. The finding of this study corroborates those studies that NRS-2002 might provide a better measure as the current criteria for nutrition risk detection.

### Strengths and limitations

This study is one of the few studies to include hospitalized adult patients with a variety of conditions that were treated both non-surgically and surgically. To our knowledge, this is the first study to evaluate the application of SGA among Chinese hospitalized patients. All the data was collected by clinical dietitians trained in PUMCH.

Selection bias in this study might lead to an underestimate of the prevalence of nutrition risk among patients in gastrointestinal department of PUMCH. Patients with critical illness, or those who were incommunicable were excluded from the present study because there was limited opportunity for interviewing. Critically ill patients might not experience the symptoms of malnutrition at the moment of assessment, but could be at a high risk of malnourishment because of the increased energy and nutrient requirements of the diseases. As a result, the high prevalence of nutrition risk demonstrated the need for including nutrition screening as a part of the admission process in Chinese hospitals, particularly among GI disease patients.

There are many factors associated with the prevalence of nutrition risk, such as social segregation, psychological factors, economic status, and lack of medical awareness [3,18], which may explain the high prevalence of nutrition risk among individuals ≤20. However, they were not measured in this study and could not be included in the analyses. Furthermore, other factors influencing clinical outcomes were not included in the data, such as the severity of disease and types of nutritional intervention provided for patients.

Because of the influences of inflammation on nutrition risk and the response to nutrition intervention and clinical outcomes [28–30], the failure of taking inflammation into consideration was a limitation of current screening tools, which should be improved in the future tool design. The 2012 consensus statement of the Academy of Nutrition and Dietetics/American Society for Parenteral and Enteral Nutrition [31] recognizes the role of inflammation in the etiology of adult malnutrition, and recommends that practitioners incorporate measures of the inflammatory response and use etiology-based diagnostic terms to improve validity of estimates of the prevalence of malnutrition in adults.

## Conclusion

The prevalence of risk of malnutrition was found to be high in gastrointestinal patients, which demonstrated the importance of including nutrition risk screening as admission evaluation.

Both NRS-2002 and SGA worked well among GI patients, and they did not perform differently for patients of different ages. There was a significant association between risk of malnutrition and clinical outcomes, but the relationship should be further explored, since many other factors including social factors, severity of diseases and nutrition intervention, influence the clinical outcomes. There was no significant difference of LOS, cost, infectious complications and death predicting capacity of NRS-2002 and SGA, however NRS-2002 performed better in predicting non-infectious complications.

## Supporting Information

S1 Dataset Dataset used in all analyses. (XLS)
